# Fluid Intake Restriction Concomitant to Sweetened Beverages Hydration Induce Kidney Damage

**DOI:** 10.1155/2020/8850266

**Published:** 2020-12-02

**Authors:** Fernando E. García-Arroyo, Edilia Tapia, Itzel Muñoz-Jiménez, Guillermo Gonzaga-Sánchez, Abraham S. Arellano-Buendía, Horacio Osorio-Alonso, Lino Manterola-Romero, Carlos A. Roncal-Jiménez, Richard J. Johnson, Laura G. Sánchez-Lozada

**Affiliations:** ^1^Dept. of Cardio-Renal Physiopathology, INC Ignacio Chávez, Mexico City, Mexico; ^2^Renal Diseases and Hypertension University of Colorado, Aurora, CO 80045, USA

## Abstract

Currently, there is the paradox of low water intake but increased intake of sugar-sweetened beverages (SB) in several populations; those habits are associated with an increased prevalence of metabolic derangements and greater chronic disease mortality. Persistent heat dehydration and increased SB intake stimulate the continued release of vasopressin and overactivation of the polyol-fructokinase pathway, synergizing each other, an effect partially mediated by oxidative stress. The objective of the present study was to evaluate whether water restriction concurrent with SB hydration can cause renal damage by stimulating similar pathways as heat dehydration. Three groups of male Wistar rats (*n* = 6) were fluid restricted; from 10 am to 12 pm animals could rehydrate with tap water (W), or sweetened beverages, one prepared with 11% of a fructose-glucose combination (SB), or with the noncaloric edulcorant stevia (ST). A normal control group of healthy rats was also studied. The animals were followed for 4 weeks. Markers of dehydration and renal damage were evaluated at the end of the study. Fluid restriction and water hydration mildly increased urine osmolality and induced a 15% fall in CrCl while increased the markers of tubular damage by NAG and KIM-1. Such changes were in association with a mild overexpression of V1a and V2 renal receptors, polyol fructokinase pathway overactivation, and increased renal oxidative stress with reduced expression of antioxidant enzymes. Hydration with SB significantly amplified those alterations, while in stevia hydrated rats, the changes were similar to the ones observed in water hydrated rats. These data suggest that current habits of hydration could be a risk factor in developing kidney damage.

## 1. Introduction

Heat stress and dehydration are accepted risk factors for developing acute kidney injury episodes that may evolve into chronic kidney disease (CKD) [1, 2]. Dehydration is a condition that increases the risk of death in adults hospitalized for respiratory illness, gastrointestinal, and cardiovascular diseases, as well as diabetes and metabolic disorders [3]. In addition, the condition defined as underhydration (low water intake or serum sodium >145 mmol/L, spot urine volume <50 mL, and/or spot urine osmolality ≥500 mmol/kg), in which total body water is maintained in normal range [4], has also been significantly associated with an increased prevalence of metabolic derangements [4]; this condition was also associated with 4.2 times greater chronic disease mortality [5]. Such data is relevant in the view that less than 50% of the women and 60% of the men do not comply with the European Food Safety Agency (EFSA) recommendations on adequate intake of water from fluids [6].

On the other hand, the current consumption of sugar-sweetened beverages (SSBs) and fruit juices have significantly increased all over the world [7]. Notably, such food items are an essential part of the western type diet [6]. Strikingly, in some countries of Latin America, over 75% of children drink at least one serving (250 mL/day) of SSBs per day, and in Mexico, 82% of adults consume more than one serving of SSBs daily [6–8]. Thus, currently, there is the paradox of low water intake but increased intake of SSBs in several populations. Significantly, the rise in SSBs intake has been related to cardiovascular disease, metabolic syndrome, cancer, hypertension, and renal damage, including kidney stones as well as all causes of death [9–12].

There is evidence that both chronic heat dehydration and increased SSBs intake stimulate similar mechanisms for the body to maintain its internal milieu. Thus, both insults induce the persistent release of vasopressin (measured as copeptin) and continue overactivation of the polyol-fructokinase pathway [13–15]. The chronic activation of both mechanisms causes the development of CKD [1, 2]. Moreover, both mechanisms synergize, resulting in the aggravation of kidney damage [16]. The interlink between both pathways was evidenced when the inhibition of vasopressin receptors with conivaptan blocked the renal overexpression of the polyol-fructokinase pathway enzymes that were in concurrence with the prevention of increased oxidative stress induced by heat dehydration plus SSB hydration [14].

Later on, it was demonstrated that oxidative stress might act as the link between vasopressin and polyol-fructokinase pathways as the supplementation with N-acetylcysteine, in combination with ascorbic acid, resulted in the prevention of renal oxidative stress and the blockade of the renal overexpression of aldose reductase, sorbitol dehydrogenase, and fructokinase, as well as with the preservation of renal function [13]. Such beneficial effects were partially mediated by the nuclear factor erythroid 2-related factor (Nrf2) nuclear translocation. Nrf2 is the master antioxidant regulator and acts by inducing the expression of antioxidant enzymes [17] when it is allowed to translocate into the cell nucleus.

Thus, we hypothesized that dehydration caused by chronic water restriction might act similar to recurrent heat-induced dehydration synergizing with SB's rehydration by stimulating similar pathways causing renal damage.

Mild chronic heat exposure *per se* can increase oxidative stress [18]. Therefore, the objective of the present study was to evaluate whether mild chronic dehydration induced by water restriction concurrent with sweetened beverage hydration can cause renal damage by stimulating vasopressin and polyol-fructokinase pathways, and by increasing renal oxidative stress.

## 2. Materials and Methods

### 2.1. Ethical Approval

The studies were conducted in adhesion with the current “Guide of Care and Use of Laboratory Animals” published by the National Institutes of Health and the Mexican Federal Regulation for the animal experimentation and care (NOM-062- ZOO-2001) and the disposal of biological residues (NOM-087-ECOL-1995) and were approved by the Instituto Nacional de Cardiología Ignacio Chavez.

### 2.2. Experimental Protocol

We included 3 groups of male Wistar rats (*n* = 6 each) that received 15 mL of tap water for 22 h/day (approximately one-third of rats regular drinking in 24 h). From 10 am and for two hours, animals could replenish with tap water (W), or sweetened beverages, one prepared with 11% of a fructose-glucose combination (SB), or with the noncaloric edulcorant stevia (ST). As rats tend to increase their fluid intake when sweetened beverages are offered, we included the stevia group for controlling the fluid volume intake. A normal control group of healthy rats was also studied (*n* = 5). The animals were followed for 4 weeks. The volume ingested during rehydration period and the solid food consumed were recorded daily. Body weight was recorded weekly.

### 2.3. Measurements

After 30 days of follow-up, rats were placed in metabolic cages (Tecniplast. Varese, Italy) by 18 hours to collect urine samples. Systolic blood pressure was measured in conscious rats with a noninvasive tail cuff and validated method [15].

Next, rats were anesthetized with isoflurane and euthanized by exsanguination of the abdominal aorta. The kidneys were washed out by perfusion with cold PBS1x, snap-frozen, and stored in liquid nitrogen until further analysis. The blood samples were centrifuged at 3500 rpm for 5 minutes, and plasma was separated and frozen at -20°C until further processing.

### 2.4. Markers of Dehydration

Plasma copeptin (a surrogate of vasopressin) was measured with an ELISA kit (Peninsula Laboratories, CA, USA) in samples previously extracted with Sep-pack cartridges C18 accordingly to manufacturer instructions (Water, Milford MA). Plasma and urine sodium concentration was determined by flame photometry. Plasma and urine osmolality were measured in an automatic freezing point osmometer (Löser Osmometer, Berlin, Germany).

### 2.5. Markers of Renal Function and Renal Tubular Damage

Plasma and urine creatinine were measured using enzymatic kits (SpinReact. Girona, Spain). With creatinine measurements, creatinine clearance (CrCl) was calculated as a proxy of the glomerular filtration rate. Urinary N-Acetyl-*β*-D-glucosaminidase (NAG) excretion was measured using 4-nitrophenyl-N-acetyl-*β*-D-glucosaminide as a substrate. The data were corrected by urinary volume as previously described [14].

### 2.6. Western Blotting Analysis

Three samples per group were randomly selected for western blot analysis. Equal amounts of protein (30 *μ*g/mL) were loaded in SDS polyacrylamide gel electrophoresis at 10% (Mini Protean II, Bio-Rad, Hercules, CA, USA). Proteins were transferred to a nitrocellulose membrane (Criterion Blotter, Bio-Rad, Hercules, CA, USA); membranes were stained with Ponceau red to confirm the transfer and blocked with nonfat dry milk (Bio-Rad, Hercules, CA, USA) 5% in TBS-tween buffer for 1 hour. Antibodies were incubated overnight at 4°C with V1a vasopressin receptor (Abcam, ab187753, 1 : 3000 dilution), V2 vasopressin receptor (Abcam, ab109326, 1 : 2500 dilution), Aldose reductase (Genetex, GTX113381, 1 : 2000 dilution), Sorbitol dehydrogenase (Genetex, GTX83588, 1 : 4000 dilution), Fructokinase (Genetex, GTX109591, 1 : 5000 dilution), Superoxide Dismutase (Santacruz Biotechnology, sc101523, 1 : 1000 dilution), Catalase (Santacruz Biotechnology, sc271358, 1 : 1500 dilution), Glutathione peroxidase (Santacruz Biotechnology, sc22145, 1 : 1000 dilution), Nox-4 (Novus Biologicals, NB-110-58851, 1 : 1000 dilution), Bcl2 (Santacruz Biotechnology, sc509, 1 : 3000 dilution), Bax (Santacruz Biotechnology, sc4239, 1 : 2000 dilution), Nrf2 (Genetex, GTX103322, 1 : 2000 dilution), and Kidney Injury Molecule (KIM-1) (Genetex, GTX85067, 1 : 3000 dilution).

To obtain nuclear extracts, renal cortex was homogenized in ice-cold buffer (10 mM HEPES, 0.2% Triton X-100, 50 mM NaCl, 0.5 mM sucrose, 0.1 mM EDTA, and protease and phosphatase inhibitors). Homogenates were centrifuged at 10,000 rpm for 10 min. The pellet was resuspended in ice-cold buffer and incubated for 15 min and centrifuged for 10 min at 14,000 rpm. The pellet was used for Nrf2 immunoblotting.

### 2.7. Intrarenal Fructose and Uric Acid

Fructose was extracted with perchloric acid and measured with the anthrone method previously described [19]. Uric acid was measured as a byproduct of fructose metabolism; it was extracted by repetitive heat-cold shock [20] from the renal cortex and was measured with a colorimetric kit (Sekisui Diagnostics, Burlington, USA). Fructose and uric acid renal contents were corrected by mg of protein measured using the method developed by Bradford.

### 2.8. Markers of Oxidative Stress

Lipid peroxidation and protein carbonylation were measured as irreversible products of oxidative stress in renal cortex homogenates with a colorimetric method previously published [21, 22]. NADHP oxidase activity was measured in renal cortex homogenates using methods previously described [23].

### 2.9. *Lactate dehydrogenase* Activity

Plasma lactate dehydrogenase was measured by an enzymatic kit (Cayman Chemical, Ann Arbor, Michigan, USA) according to manufacturer instructions.

### 2.10. Fructokinase Activity

Fructokinase activity was measured in renal cortex homogenates by a method previously reported [24].

### 2.11. Statistical Analysis

Results are presented as mean ± SD and analyzed by one-way ANOVA followed by Tukey's multiple comparison test. Statistical significance was established as ^∗^*p* ≤ 0.05, ^∗∗^*p* ≤ 0.01, ^∗∗∗^*p* ≤ 0.001, and ^∗∗∗∗^*p* ≤ 0.0001.

## 3. Results

### 3.1. Water, Food Consumption, and Body Weight

Rats that received tap water ingested 8 ± 1 mL (mean) during the rehydration period (two hours each day). The groups that received sweetened waters, either as stevia or SB, consumed similar mean amounts of water for rehydration (16 ± 2 and 16 ± 2 mL, respectively), which represented 50% more volume, compared to tap water group (Table 1). All rats consumed the 15 mL of water left during the 22 h of fluid restriction. On the other hand, animals rehydrated with SB water consumed less solid food compared to stevia and water; this trend was found to be significant (Table 1). Nevertheless, total daily caloric intake, provided by solid food plus fluids, was comparable among the four groups (control 80.2 ± 3, 2 kcal/day; water 76.3 ± 9.61 kcal/day, SB: 78.6 ± 5.51 kcal/day and Stevia 75.4 ± 6.75 kcal/day), and weight gain was not different among the groups (Table 1).

### 3.2. Fluid Restriction and Hydration with SB-Induced Mild Dehydration, Renal Functional Alterations, Mild Hypertension, and Induced Tubular Damage

**Figure 1:**
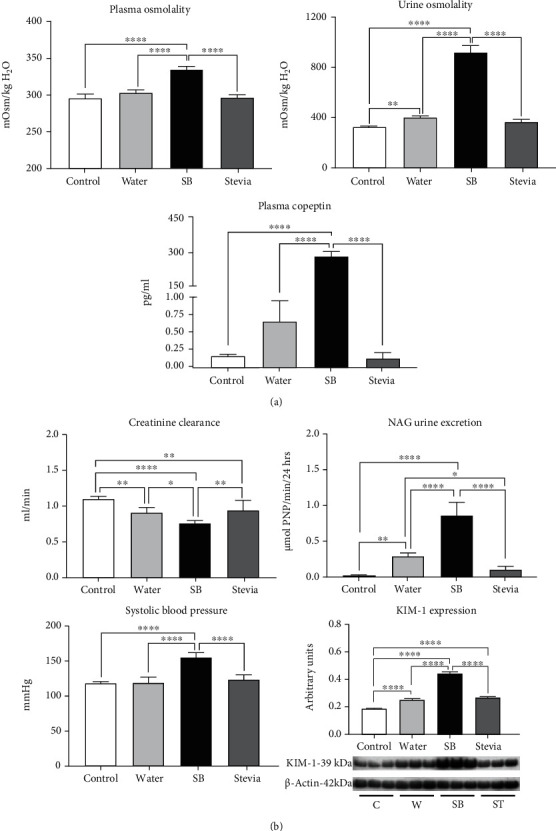
Effects of water restriction and rehydration with a SB on hydration and renal function markers. (a) Markers of hydration: plasma and urine osmolality and plasma copeptin. (b) Markers of renal damage (creatinine clearance, NAG urine excretion, and KIM-1 renal cortex expression) and systolic blood pressure. For western blotting, 3 randomly selected samples per group were analyzed. Results are presented as mean ± SD and analyzed by one-way ANOVA followed by Tukey's multiple comparison test. Statistical significance was established as ^∗^*p* ≤ 0.05, ^∗∗^*p* ≤ 0.01, ^∗∗∗^*p* ≤ 0.001, and ^∗∗∗∗^*p* ≤ 0.0001.

After 22 hours of fluid restriction and hydration with tap water by 2 hours, there was a mild but significant increment in urine osmolality and an increase of 4 times in plasma copeptin in comparison to control rats. On the other hand, hydration with SB in fluid-restricted rats induced a further increment in plasma and urinary osmolality and plasma copeptin concentration compared vs. C, W, and ST groups (Figure 1(a)). Fluid restriction plus water hydration also induced mild renal alterations as CrCl fell by 15% and the markers of tubular damage NAG and KIM-1 mildly but significantly increased. On the other hand, rehydration with SB further reduced creatinine clearance and further increased NAG and KIM-1 (Figure 1(b)). In addition, fluid restriction plus hydration with SB increased systolic blood pressure (SBP) (Figure 1(b)). Fluid-restricted rats hydrated with stevia behaved similarly to W group at all these parameters.

### 3.3. Vasopressin and Polyol-Fructokinase Pathways Were Overactivated by Fluid Restriction and Hydration with SB

**Figure 2:**
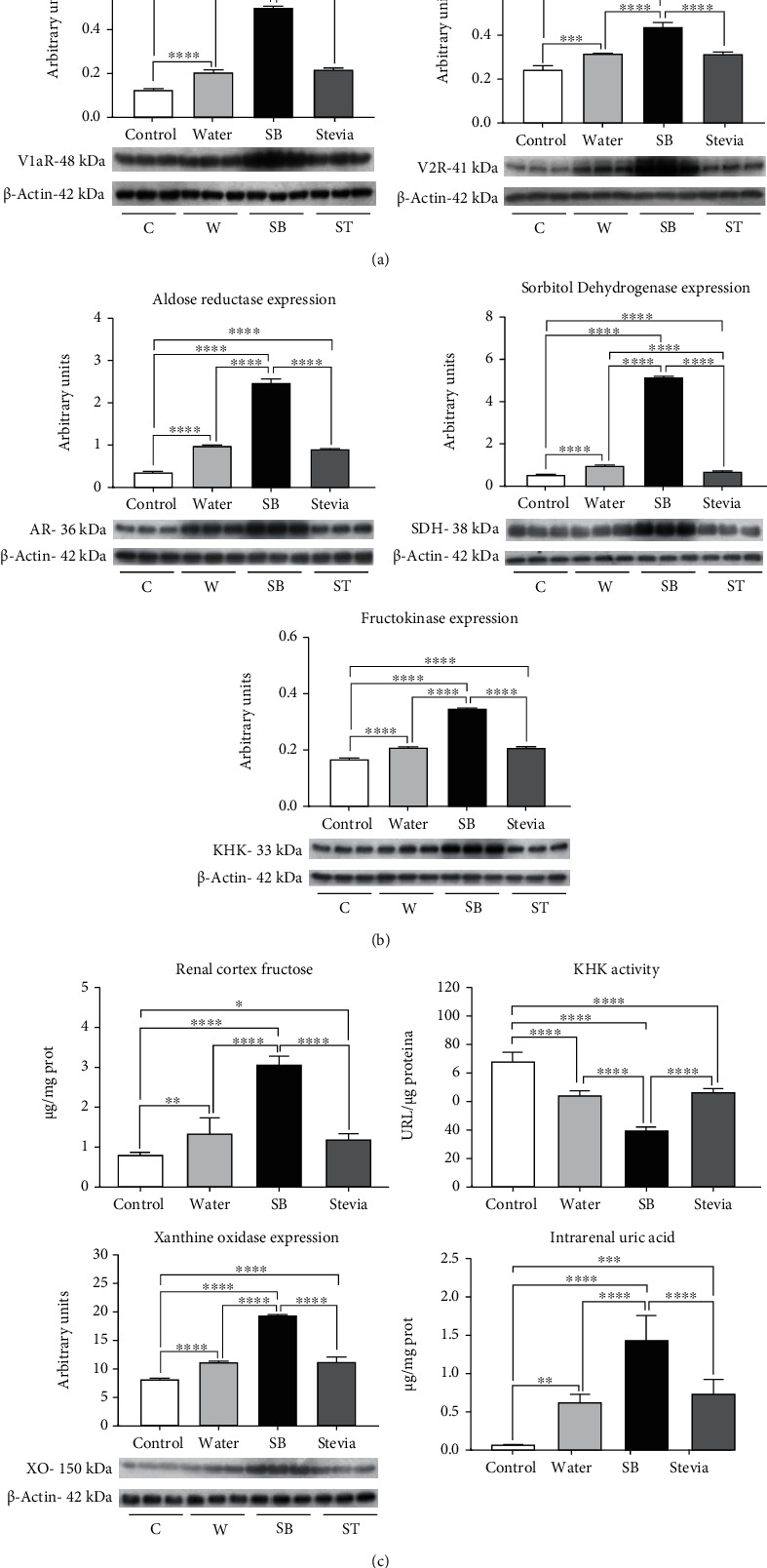
Renal expression of renal vasopressin receptors and polyol-fructokinase pathway enzymes. (a) V1a and V2 vasopressin receptors expression in renal cortex. (b) Aldose reductase, sorbitol dehydrogenase, and fructokinase (KHK) expression in renal cortex. (c) Renal cortex fructose and uric acid concentrations, KHK activity, and xanthine oxidase expression. For western blotting, 3 randomly selected samples per group were analyzed. Results are presented as mean ± SD and analyzed by one-way ANOVA followed by Tukey's multiple comparison test. Statistical significance was established as ^∗^*p* ≤ 0.05, ^∗∗^*p* ≤ 0.01, ^∗∗∗^*p* ≤ 0.001, and ^∗∗∗∗^*p* ≤ 0.0001.

Vasopressin and polyol-fructokinase pathways with the concomitant synthesis of endogenous fructose and uric acid are mechanisms activated by heat stress-induced dehydration. In the present study, we observed that dehydration induced by water restriction also activated such mechanisms (Figures 2(a)–2(b)). Fluid-restricted rats hydrated with water had a mild but significantly higher expression of renal cortex V1a and V2 vasopressin receptors, aldose reductase, sorbitol dehydrogenase, fructokinase, and as well as a higher concentration of renal fructose (Figures 2(a)–2(b)). As fructokinase plays a key role in the renal damage induced by dehydration [24], we indirectly evaluated its activity. In fluid-restricted rats hydrated with water, there was a mild increment in KHK activity (lower ATP tissue concentration). In addition, XO expression, as well as the by-product of fructose metabolism, UA, was also increased in this group (Figure 2(c)).

Previously it was shown that in heat-dehydrated rats, such mechanisms were further overactivated when rats were hydrated with SB [25, 26]. In the present study, we found a similar effect in fluid-restricted rats. Therefore, we found an additional overexpression in vasopressin receptors and polyol-fructokinase enzymes and fructose renal cortex concentrations. Furthermore, KHK activity was further increased as well as the expression of XO and UA renal cortex concentrations. On the other hand, fluid-restricted rats hydrated with stevia showed a similar effect as water hydrated rats (Figure 2(c)).

### 3.4. Renal Oxidative Stress and Apoptosis Were Increased by Fluid Restriction and Rehydration with Sweetened Beverages

**Figure 3:**
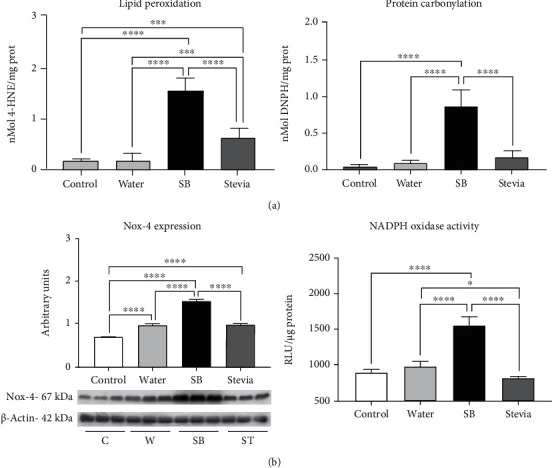
Oxidative stress in renal cortex. (a) Renal cortex lipid peroxidation measured as 4-hydroxynonenal adducts and protein carbonylation. (b) Renal cortex Nox-4 expression and NADPH oxidase activity. For western blotting, 3 randomly selected samples per group were analyzed. Results are presented as mean ± SD and analyzed by one-way ANOVA followed by Tukey's multiple comparison test. Statistical significance was established as ^∗^*p* ≤ 0.05, ^∗∗^*p* ≤ 0.01, ^∗∗∗^*p* ≤ 0.001, and ^∗∗∗∗^*p* ≤ 0.0001.

**Figure 4:**
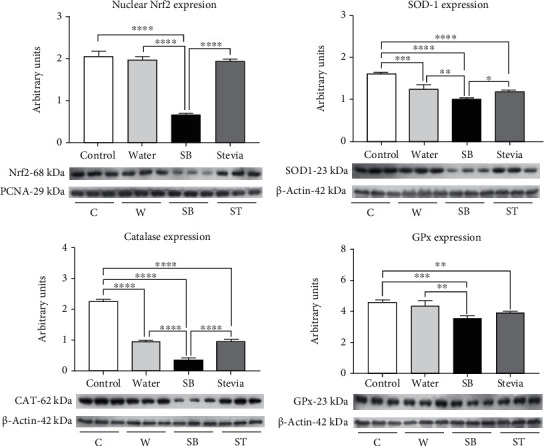
Antioxidant enzymes renal expression and Nrf2 nuclear translocation. Renal expression of superoxide dismutase, catalase, and glutathione peroxidase. Nuclear translocation of Nrf2 on renal cortex homogenates. For western blotting, 3 randomly selected samples per group were analyzed. Results are presented as mean ± SD and analyzed by one-way ANOVA followed by Tukey's multiple comparison test. Statistical significance was established as ^∗^*p* ≤ 0.05, ^∗∗^*p* ≤ 0.01, ^∗∗∗^*p* ≤ 0.001, and ^∗∗∗∗^*p* ≤ 0.0001.

Lipid peroxidation and protein carbonylation were measured in the renal cortex as markers of oxidative stress. Fluid restriction and hydration with water did not induce an increment of these markers of oxidative stress in comparison to the control group (Figure 3(a)). Despite NOX-4 expression was mildly increased in W in comparison to C, NADPH oxidase activity remained similar between these two groups. (Figure 3(b)). In agreement with such findings, Nrf2 nuclear translocation (the master regulator of the antioxidant response) in W group was like C group; however, SOD-1 and catalase expressions were significantly reduced, while GPX remained without changes in W rats. (Figure 4).

On the other hand, fluid restriction plus SB hydration increased oxidative stress markers by 15 times, and this was in concurrence with NOX-4 overexpression and a significant increment of NADPH oxidase activity (Figure 3(b)). In this group, we observed a significantly decreased nuclear translocation of Nrf2 in concert with a significant reduction in the expression of catalase, SOD-1, and GPx (Figure 4). Finally, hydration with stevia in fluid-restricted rats produced mild oxidative stress accompanied by mildly but significantly higher NOX-4 expression but preserved NADPH oxidase activity (Figure 3(b)) and Nrf2 nuclear translocation, despite SOD-1, catalase, and Gpx expressions were significantly reduced.

**Figure 5:**
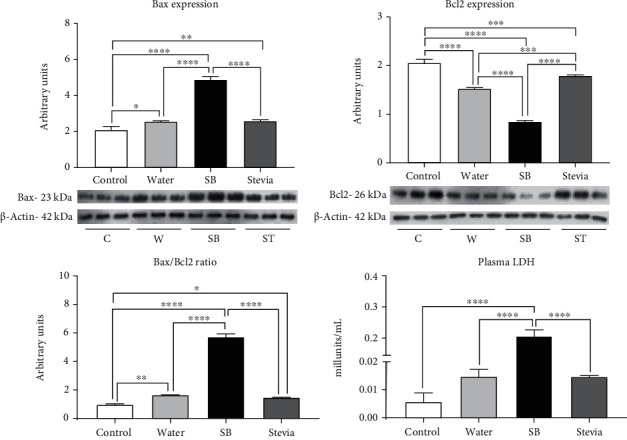
Apoptosis markers. Renal cortex Bax and Bcl2 expressions and Bax/Bcl2 ratio. Plasma LDH concentrations. For western blotting, 3 randomly selected samples per group were analyzed. The results are presented as mean ± SD and analyzed by one-way ANOVA followed by Tukey's multiple comparison test. Statistical significance was established as ^∗^*p* ≤ 0.05, ^∗∗^*p* ≤ 0.01, ^∗∗∗^*p* ≤ 0.001, and ^∗∗∗∗^*p* ≤ 0.0001.

Apoptosis is a secondary effect of Nox4 activation and oxidative stress in tubular cells [27]. Fluid restriction plus water hydration induced a mild but significant increase in Bax expression and a significant decrement in Bcl2 expression suggesting renal cortex apoptosis in this group as indicated by the significant increment in Bax/Bcl2 ratio; nevertheless, plasma LDH, a general marker of apoptosis, had a nonsignificant increment (Figure 5). On the other hand, fluid restriction followed by SB hydration induced a further overexpression of Bax and a reduction in the expression of Bcl2 of 50% and increased by three times Bax/Bcl2 ratio in comparison to W group. Accordingly, plasma LDH was twice greater in comparison to W group. Stevia hydrated group showed a similar behavior as W hydrated group (Figure 5).

## 4. Discussion

In the present work, we demonstrated that intermittent fluid restriction caused mild renal damage prompted by the mechanisms that were previously reported in heat-dehydrated rats. Thus, fluid restriction upregulated the vasopressin renal receptors, overexpressed the renal polyol-fructokinase pathway enzymes, and increased intrarenal oxidative stress. We also showed that in fluid restriction, SB hydration also exacerbates such mechanisms. Therefore, these data suggest that poor hydration habits might be a risk factor for developing kidney damage.

In our study, water restriction and intake of SB as fluid of rehydration induced a mild dehydration state similar to heat stress models [15, 28] with a significant increase in vasopressin secretion (measured as plasma copeptin) as well as a robust stimulation of renal vasopressin receptors and polyol-fructokinase pathway enzymes including increased KHK activity [24, 29]. Interestingly, rats receiving tap water during rehydration drank much less water but still had a lower plasma copeptin concentration as well as a mild upregulation of renal vasopressin receptors and polyol-fructokinase enzymes and KHK activity. On the other hand, rats hydrated with stevia, which had a similar fluid intake as the SB group, had similar responses as the rats hydrated with tap water. Therefore, these findings document that it is not simply how much rehydration fluid is administered, but that the type of fluid also matters as it relates to the regulation of vasopressin and polyol-fructokinase pathways.

Kidney alterations were in parallel to the level of upregulation of vasopressin and polyol fructokinase pathways. Therefore, water restriction and rehydration with a sweetened beverage induced a significant fall in CrCl and the upregulation of tubular damage markers (KIM-1 and NAG). Our data suggest that the potential mediator between these effects is a concurrent increase of intrarenal oxidative stress. In this regard, increased renal oxidative stress has been associated with kidney damage induced by heat stress models [14, 15] as well as other models of kidney damage [20]. In the present study, we observed that the level of activation of the polyol-fructokinase pathway was associated with an increased expression of XO and uric acid concentrations in the renal cortex. Uric acid is a prooxidant molecule that induces kidney damage [30, 31] through the activation of NOX-4. In effect, in the present study, we observed that Nox-4 was upregulated, and NADPH oxidase renal activity increased, effects better observed in the fluid restricted plus hydration with SB group. This prooxidant effect was supported by a significant increase in the tissue markers of lipid peroxidation and protein carbonylation. In previous studies from our group, we showed that heat stress and hydration with fructose-containing beverages induce the overexpression of Nox-4 as well as the catalytic accompanying subunits p22 and Gp-91 [14]. In concert with a prooxidative milieu caused by fluid restriction and SB hydration, we also found a significant reduction of the antioxidant system. Therefore, SOD, CAT, and GPx expressions were significantly decreased in the fluid-restricted group hydrated with SB. Such an effect partially resulted from a decreased nuclear translocation of Nrf2, which is their principal transcription factor. Another detrimental effect of Nox-4-mediated oxidative stress is to promote apoptosis in proximal tubule cells [27], a secondary effect of an imbalance of Bax/Bcl-2 [27]. In this regard, mitochondrial damage induced by uric acid in renal cells also is associated with an increased apoptosis [32]. In the present study, we found an imbalance of Bax and Bcl2 expressions related to more significant oxidative stress in the fluid-restricted plus SB hydrated group, suggesting an increase in renal apoptosis. This finding was further supported by an increase in LDH activity in the plasma samples from the same group.

Some limitations of this study should be stated. This is an experimental study in which the water restriction and the hydration beverages content were variables fully controlled. Therefore, this is somehow different from the situation found in free-living individuals. As such, the findings cannot be immediately extended to humans.

## 5. Conclusions

In summary, the chronic fluid restriction can induce mild renal alterations that are aggravated when a sweetened beverage is used as hydration fluid. The kidney damage was partially mediated by increased vasopressin release, overactivation of the polyol-fructokinase pathway, increased oxidative stress, and apoptosis. These data suggest that current habits of hydration could be a risk factor in developing kidney damage.

## Figures and Tables

**Table 1:** Body weight and mean food, fluid, and caloric intake after 30 days of follow-up. Results are presented as mean ± SD and analyzed by one-way ANOVA followed by Tukey's multiple comparison test. Statistical significance was established as ^**a**^*p* ≤ 0.05 vs. C, ^**b**^*p* ≤ 0.01 vs. W, and ^**c**^*p* ≤ 0.001 vs. SB.

Group/parameter	Control	Water	Sweetened beverage	Stevia
Body weight (g)	229.6 ± 4.7	239.2 ± 4.5	233.8 ± 3.5	233 ± 4.1
Food intake in 24 h (g)	24.1 ± 4	23.1 ± 2	19.7 ± 2^**a,b**^	22.8 ± 2^**c**^
Food calories (kcal/day)	80.2 ± 3.2	76.3 ± 6.8	65.1 ± 5.7^**a,b**^	75.4 ± 6.8^**c**^
Beverage intake in 2 h rehydration (mL)	—	8 ± 1	16 ± 2^**b**^	16 ± 2^**b**^
Beverages calories in 2 h rehydration (mL)	—	—	13.5 ± 5.4	—
Total fluid intake (mL)	29 ± 0.3	23 ± 1.6^**a**^	31 ± 2.8^**b**^	31 ± 2.4^**b**^
Total calories intake (kcal/day)	80.2 ± 3.2	76.3 ± 6.8	78.6 ± 5.5	75.4 ± 6.8

## Data Availability

Data is available from the corresponding author upon request.
